# Co-creation as a knowledge mobilization process in digital health regulation in East Africa: The making of the DigiReg compass

**DOI:** 10.1186/s12961-026-01488-z

**Published:** 2026-05-06

**Authors:** Sharifah Sekalala, Shajoe J. Lake, Paul Mbaka, Ryan Nyotu, Emmanuel Muhoza, Alikher Samow, Francis Mbate

**Affiliations:** 1 Centre for Global Health Law, University of Warwick, Coventry, CV4 7AL UK; 2 Division of Health Information, Ministry of Health, Kampala, Uganda; 3 Ministry of Health, Nairobi, Kenya; 4 Ministry of Health, Kigali, Rwanda; 5 Office of the Data Protection Commissioner, Nairobi, Kenya; 6 Africa CDC, Addis Ababa, Ethiopia

**Keywords:** Digital health, Health systems, East Africa, Regulation, Health policy

## Abstract

Regulating digital health in low- and middle-income countries requires policy-makers to organize and use multiple forms of knowledge, including academic research, technical expertise and experiential insights, to make tough decisions under conditions of existential uncertainty. We examine co-creation as a mode of knowledge mobilization in digital health regulation in Kenya, Rwanda and Uganda. Using content analysis and a multi-country stakeholder workshop, we trace how regulators, clinicians, technologists and civil society actors collectively interpreted comparative evidence on existing frameworks and used it to negotiate trade-offs in the institutional design of digital health regulatory frameworks. Through this process, participants co-produced the DigiReg Compass, a tiered heuristic that structures options for legal form, administrative arrangements, expertise, financing, participation and political sponsorship. We show, first, how co-creation can extend regulatory capacity by organizing distributed expertise into a usable evidence base; second, how social learning in such settings reshapes actors’ understandings of regulatory problems and solutions; and third, how tools such as the DigiReg Compass can function as knowledge translation mechanisms that link health systems research to regulatory practice. These findings contribute to debates on the organization and use of health research in health systems by illustrating how, in resource-constrained contexts, participatory processes can transform fragmented knowledge into shared guidance for regulatory decision-making.

## Background

### East Africa: A hotbed for digital health innovation

East Africa has become a hotbed for digital health innovation due to technological shifts that have turned countries such as Kenya and Uganda into testbeds for new ways of organizing care. The rapid spread of mobile phones and mobile internet, alongside fibre-optic investments since the 2000s, created dense digital infrastructures that states, donors and firms have used to pilot health platforms at scale [21]. Many of these initiatives borrow directly from mobile money innovations such as M-PESA, which showed how phones could facilitate payments and has since inspired health wallet and insurance-linked products that blur the line between healthcare and finance and become embedded into everyday life [7, 19, 21]. These innovations generate proprietary datasets, technical architectures and business models that are often scaled and monetized that are by firms and investors headquartered in high-income countries, with few guarantees that informational or financial value circulates back into East African public health systems [21, 26].

At the core of public health information systems in countries such as Kenya and Uganda are open-source platforms such as DHIS2 and OpenMRS, which underpin routine reporting and electronic medical records and are identified as key building blocks of national digital health strategies across Africa [35]. In Kenya, for example, the WelTel Kenya1 trial demonstrated that a simple weekly two-way Short Message Service (SMS) between clinic staff and patients could significantly improve adherence and viral suppression for people living with human immunodeficiency virus (HIV), and it has since become an emblematic example of low-cost mobile health at a national scale [16]. Building on this infrastructure, newer private platforms have emerged within the East African digital health ecosystem. African Digital Health, Rocket Health in Uganda and Kenyan ventures such as MyDawa and Zuri Health combine telemedicine, online pharmacy, laboratory services and logistics and are positioned by investors and development partners as flagship models of digitally enabled primary care in the region [23, 28]. Within this commercial space, decisions about platform design, benefit structures and data governance are increasingly shaped by corporate actors, situating digital health within broader debates on adverse commercial determinants of health in low- and middle-income countries (LMIC) [26].

### Regulation outpaces digital health innovation

The rapid spread of digital tools, especially in low- and middle-income countries (LMICs), however, outpaces the development of appropriate governance arrangements, producing fragmented, siloed initiatives instead of integrated digital health systems. Multiple ministries, regulators and development partners introduce overlapping programmes, standards and platforms, often with limited national stewardship and coordination [34]. New actors such as technology firms, venture-backed start-ups and global philanthropies are increasingly shaping core health system functions previously led by public authorities, raising questions about accountability and control [21, 26]. These questions extend beyond classical regulatory capacity to encompass deeper questions on who controls and benefits from the economic and informational benefits of digital health infrastructures, and to what extent LMIC governments can constrain extractive business models that treat health data and patient engagement as assets for global markets?

Many of the existing legal and regulatory frameworks were designed for paper-based information flows and vertically organized service delivery and are ill-suited to crosscutting, data-intensive digital infrastructures (World Health Organization 2021). The result is a patchwork of rules, guidelines, contracts and informal practices that are difficult to coordinate and even harder to adapt as technologies evolve and involve more corporate actors in a field that was primarily regulated by the state. One particularly acute dimension of this governance gap concerns data. Every digital health platform generates large volumes of personal and sensitive information about people and their health, amplifying concerns about cybersecurity, secondary use and data exploitation. In practice, digital health programmes in East Africa frequently feed into transnational data value chains, where anonymized or pseudonymized, datasets are used to train algorithms, refine insurance and credit products or support analytics firms that are commonly based in high-income jurisdictions [20, 26].

In East Africa, statutory reforms have begun to respond to these concerns but in uneven ways. In Kenya, the Data Protection Act, 2019, has been criticized as rushed and insufficient to protect citizens’ data in a rapidly financialized and digitalizing health landscape (prompting subsequent initiatives such as the Digital Health Act, 2023, which seeks to provide a more comprehensive framework for health data governance [25]. Tanzania has similarly faced criticism for weak protection of digital data and only recently adopted the Personal Data Protection Act, 2022, establishing a general framework for regulating personal and health data [29]. Rwanda and Uganda have enacted cross-cutting data protection laws that cover health data [11, 12], but the digital dimension is still largely addressed through sectoral instruments, such as e-health policies and national digital health guidelines rather than consolidated, dedicated digital health statutes [25]. These developments expand the formal regulatory space, but they do not on their own resolve how data are used, by whom, and for what purposes. Our concern in this paper aligns with these critiques by examining how regulators in Kenya, Rwanda and Uganda attempt to organize knowledge and institutionalize co-creation to retain public oversight over commercially driven digital health systems.

In practice we know that regulators and policy-makers make decisions under conditions of profound uncertainty as evidence on the effectiveness, safety and equity impacts of digital health tools remains limited, unevenly distributed and often contextually thin [33]. Official statistics, routine health information systems and research studies provide only partial snapshots of digital health in practice, with persistent gaps around real-world implementation, interoperability and outcomes Frontline providers, patients, civil society actors and innovators therefore hold crucial experiential knowledge about how tools are actually used, what risks they generate and which safeguards matter most, yet these perspectives are frequently under-represented in formal evidence bases [21]. Additionally, this knowledge is also fragmented across organizations and initiatives and is rarely assembled into a coherent foundation for regulatory decision-making, a gap that recent work on knowledge mobilization and digital-in-health explicitly seeks to address [9]. Many LMIC regulatory bodies thus “learn to steer while building the ship.” They must design and implement oversight mechanisms at the same time digital health systems are being constructed. The practical question for them is less “What is the ideal regulatory model?” and more “How can governments organize and use diverse forms of knowledge (research evidence, routine data, professional expertise and lived experience) to make credible decisions about digital health under severe capacity constraints?”.

Existing accounts of evidence use in health policy have paid limited attention to these regulatory settings, especially in relation to digital health in East Africa. We argue that one promising answer lies in co-creation processes that are explicitly oriented towards knowledge mobilization, understood as the active, iterative and collaborative generation, sharing and use of evidence in decision-making [13]. By co-creation we mean structured, iterative engagements in which regulators, policy-makers, providers, technologists and civil society actors collectively interrogate available evidence, surface experiential insights and negotiate trade-offs in regulatory design – an approach increasingly used to mobilize knowledge in health systems and public health [13]. Co-creation is a practical response to the reality that no single actor holds all the relevant knowledge, and that capacity can be built through organized interaction as much as through formal training or institutional reform [17].

We examine a multi-country co-creation process on digital health regulation in Kenya, Rwanda and Uganda, focusing on how it organized and mobilized knowledge for regulatory decision-making. We analyse how documentary evidence from existing laws, policies and strategies was assembled into comparative inputs; how stakeholders used and contested this material; and how experiential knowledge from their respective systems informed the design of regulatory options. The process culminated in the co-creation of the DigiReg Compass, a tiered heuristic that structures regulatory choices across domains, such as legal form, institutional arrangements, expertise, financing, participation and political sponsorship, which we treat not only as a product of co-creation but as a knowledge translation tool that crystallizes and makes reusable the learning generated through the process.

We make three original contributions. First, we advance a conceptualization of co-creation in digital health regulation as a mode of knowledge mobilization – a way of organizing how research evidence, routine data and experiential insights are brought into conversation to shape regulatory choices in capacity-constrained settings. Second, we provide an empirical account of how regulators and other stakeholders in Kenya, Rwanda and Uganda engaged with comparative documentary evidence and with one another’s situated expertise to rethink problems and options for digital health oversight. Third, we introduce the DigiReg Compass as a practical device for structuring regulatory decision-making, showing how such tools can capture and carry forward the knowledge generated in co-creation processes.

## Methods

### Research design

To examine co-creation as a mode of knowledge mobilization for digital health regulation, we adopted a qualitative, multi-country case study design. This design was suited to capturing the complex, context-dependent dynamics of regulatory decision-making in Kenya, Rwanda and Uganda and to studying co-creation as both an empirical process and an analytic lens. The study comprised two linked components: (i) a content analysis of laws, policies and strategies to establish a comparative baseline of existing institutional arrangements and (ii) a participatory stakeholder workshop, with focus group discussions, which operationalized co-creation by engaging regulators, policy-makers and other actors in collective interpretation of this material and in the joint development of regulatory options. These components enabled us to examine not only how regulation is configured on paper but also how stakeholders organize, contest and generate knowledge through interaction and cross-country comparison.

### Analytical framework

We situate co-creation at the intersection of participatory governance, social learning and knowledge mobilization in health systems. Collaborative governance literature shows how governments increasingly convene diverse actors to address complex policy problems that no single organization can solve [8]. Recent work on public governance as co-creation extends this by treating multi-actor collaboration not only as a democratic aspiration but as a way of generating public value and innovation under conditions of uncertainty [1]. In parallel, the knowledge mobilization literature highlights the need for active, relational processes that move knowledge across organizational and professional boundaries, rather than assuming research findings diffuse automatically into policy and practice [13]. In digital health, where evidence is fragmented and expertise widely distributed, these strands converge as governance depends on organizing how regulators, health workers, technologists, patients and communities generate, share and use knowledge together [15].

Within this broader field, scholars distinguish co-production from co-creation. Co-production typically refers to service users and professionals working together to tailor or deliver services within largely fixed institutional arrangements [5]. Co-creation, by contrast, involves a broader set of actors, such as regulators, providers, innovators and community groups, working in structured processes that give them influence over problem definition, design choices and sometimes implementation [1]. In health, co-approaches have increasingly been used to mobilize knowledge by combining research-based, professional and experiential insights in the management of conditions and the design of interventions [13]. We adopt this latter understanding of co-creation as a multi-actor, iterative process explicitly oriented towards bringing different forms of evidence and experience into conversation and turning them into shared guidance for action.

The central rationale for co-creation in LMIC digital health regulation is therefore epistemic. Social learning research emphasizes how sustained interaction in heterogeneous groups enables actors to revise their understandings, preferences and assumptions, thereby enhancing collective problem-solving capacity [14]. Co-creation institutionalizes such interaction through structured forums where regulators, innovators, professionals and citizens examine documentary evidence, surface tacit knowledge and negotiate what counts as a problem and a viable solution [18]. In doing so, co-creation can support both single-loop adjustments to existing rules and double-loop questioning of underlying goals and framings while simultaneously building confidence and capability to interpret and apply evidence [10].

Yet, co-creation can be understood as a procedural innovation in governance, particularly suited to fast-moving digital health contexts. Experimentalist and collaborative innovation perspectives conceptualize regulation as a provisional, revisable arrangement, updated through cycles of experimentation, feedback and peer review rather than fixed once and for all [24, 27]. Emerging practices in digital health such as stakeholder dialogues, sandboxes and iterative rulemaking exemplify this shift towards agile, learning-oriented regulation [15]. Recent work argues that inclusive co-creation should become a default mode of digital health innovation and governance, with regulators, clinicians, patients and industry co-designing both technologies and oversight mechanisms [4].

The promise of co-creation is tempered by significant practical and political challenges. Implementing co-creation demands time, resources, careful facilitation and organizational commitment, all of which are often scarce in LMIC regulatory agencies [18]. Power asymmetries can distort whose knowledge is heard. Global firms and donors may dominate agendas, while communities or frontline workers struggle to influence outcomes [22]. Digital-health-specific perspectives similarly caution that, without deliberate attention to inclusion, co-creation risks reproducing existing inequities in who designs innovations and who bears their risks [4]. In our account, co-creation is valuable not because it produces consensus, but because it creates a collaborative space in which competing problem framings, interests and forms of expertise can be openly contested and worked on together. Our framework therefore positions co-creation as both a learning mechanism and a contested site where knowledge claims are negotiated. It treats regulation as an adaptive process whose quality depends on how effectively diverse forms of knowledge are organized, questioned and translated into institutional practice, and on whether the conditions for meaningful, equitable participation can be secured. For us, disagreement and contestation are not seen as failures of co-creation but are primary mechanisms through which participants test claims, surface tacit assumptions and renegotiate shared understandings.

### Case selection

We selected Kenya, Rwanda and Uganda as comparative cases. All three are East African LMICs that have recently advanced digital health initiatives but exhibit divergent regulatory approaches. Kenya has pursued formalized legislation, including a Digital Health Act and a semi-autonomous Digital Health Agency, backed by strong political sponsorship; Rwanda has emphasized inter-ministerial coordination and national e-health policies; and Uganda, while home to a dynamic e-health ecosystem, relies largely on policies and guidelines and is in the early stages of developing formal regulation. This variation provided fertile ground for cross-case learning, enabling participants to compare experiences ranging from statutory frameworks to more flexible, distributed arrangements. In each country, digital health ecosystems are active and evolving, encompassing mobile health (mHealth) programmes, telemedicine pilots and data-driven initiatives, making regulatory design both timely and salient.

### Data collection

We used a two-phase strategy.

Phase 1: Content analysis. We conducted content analysis of eight policy and regulatory documents across the three countries, including Kenya’s Digital Health Act and strategy, Rwanda’s Health Information and Communication Technology (ICT) guidelines and Uganda’s eHealth policy. These eight documents were selected because they constituted the primary domestic level instruments governing digital health at the time of study in each country, and they were also widely referenced by stakeholders as structuring the digital health landscape. Here we draw on public administration work that foregrounds institutional design choices around legal form, organizational location, expertise, financing, participation and political sponsorship as key levers in configuring state capacity (Goodin 1996) [1].

**Fig. 1:**
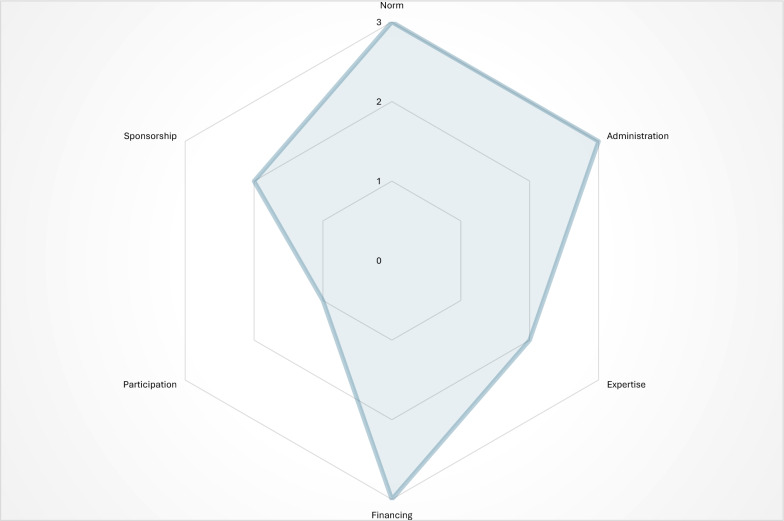
Institutional design of Kenya’s digital health regulatory framework

**Fig. 2:**
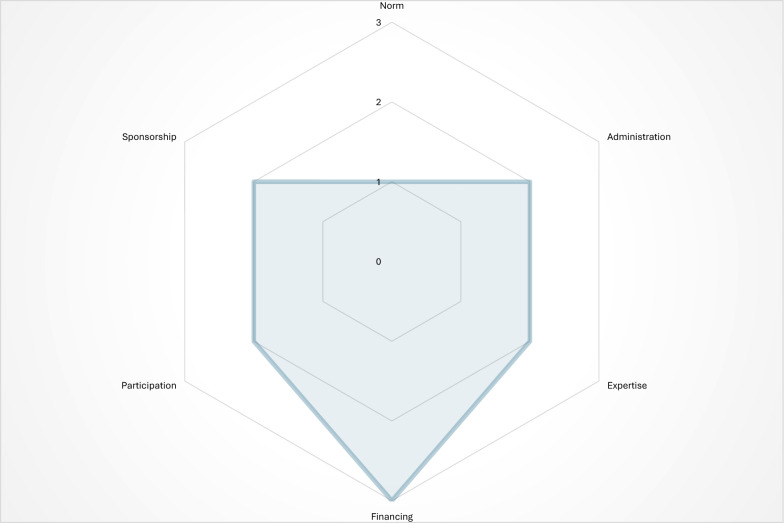
Institutional design of Rwanda’s digital health regulatory framework

**Fig. 3:**
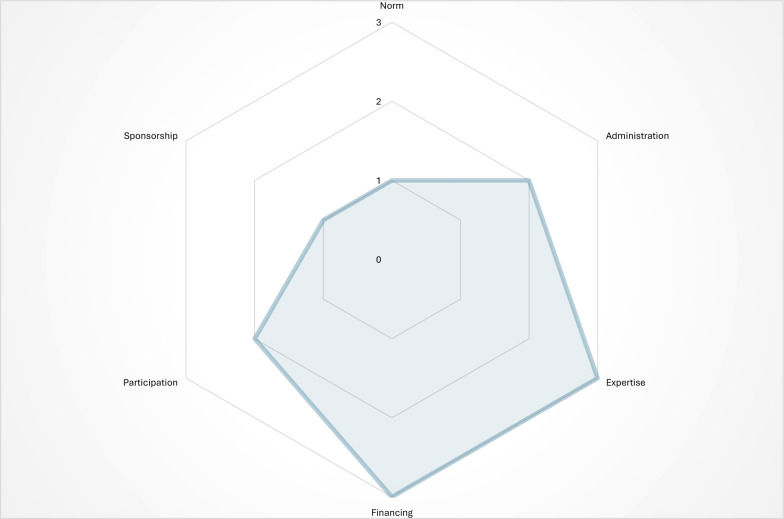
Institutional design of Uganda’s digital health regulatory framework

We coded the documents against six institutional design features to generate a draft evaluative framework for digital health regulation, including legal basis, administrative structures, inclusion of expertise, financing mechanisms, stakeholder participation and political sponsorship (Table 1), which we then used as a starting point for the Phase 2 co-creation workshop. This established a comparative baseline, illustrating differences such as Kenya’s reliance on statutory law versus Uganda’s policy-based approach, or Rwanda’s inter-ministerial task force versus Uganda’s intra-ministry team (Figs. 1, 2 and 3).

**Table 1:** Tiers within each design feature

Feature	Tier 1	Tier 2	Tier 3
Norm	Policies, guidelines	Hybrid	Statute
Administration	Internal	Inter-ministerial	External agency
Expertise	Internal	External	Hybrid
Financing	Existing	Redistributed	Budgetary
Participation	Laissez-faire	Ad hoc	Integrated
Sponsor	Junior	Senior	Political

Phase 2: Stakeholder co-creation workshop. We convened a 2-day workshop in Kampala, Uganda, with 21 participants representing ministries of health and ICT, data protection authorities, clinicians, nongovernmental organizations (NGOs), academics, private-sector innovators and civil society (Table 2). The workshop combined eight plenary sessions, reviewing the content analysis and draft framework derived from Phase 1, with four focused breakout discussions, exploring each design feature in depth. Semi-structured guides prompted deliberation on trade-offs (for example, legislation versus guidelines) and stakeholder roles. All sessions were transcribed, with facilitators recording detailed notes on both content and dynamics. Facilitators used a simple discussion guide that prompted them to record (i) key substantive points, (ii) moments of disagreement or apparent learning and (iii) observations on participation dynamics, such as who spoke, who responded and how power relations appeared to shape the discussion. In designing and facilitating the workshop, we paid attention to what circumstances created conditions for candid reflection and mutual learning, from the language used in invitations, which framed participation as joint inquiry as opposed to validation, to acknowledgement of institutional limits and openness about uncertainty. Small, mixed-stakeholder breakout groups were used to lower status barriers, while plenary sessions began with regulators reflecting on their own frameworks before others commented, to normalize self-critique.

**Table 2:** Stakeholders’ characteristics

Participants in simulation by sector	
**Civil society**	□□□□
**Academia**	□□□
Law	□
Innovation	□
Social science	□
**Media**	□□
**International institutions**	□
**Policy-makers**	□□□□□□□
**Innovators**	□□
**Regulators**	□□

Each box represents one participant

### The co-creation process

The workshop centred on the iterative development of the DigiReg Compass, a regulatory design heuristic and knowledge translation tool. Drawing on Phase 1, we presented an initial draft evaluative framework, structured around six institutional design features in tiered form – (1) norms, (2) administration, (3) expertise, (4) financing, (5) participation and (6) sponsorship (Table 3) – as the basis for plenary discussion and subsequent revision into what became the DigiReg Compass. In plenary sessions, this framework was used both to present comparative documentary evidence and to structure discussion, prompting participants to locate their national arrangements within the tiers and to reflect on their implications. We made a central design choice to foreground contestation by explicitly inviting participants to challenge the documentary analysis, question the framing of tiers in the Compass and dispute each other’s interpretations.

**Table 3:** Initial evaluative framework for conducting the content analysis

Domain	Meaning
Accountability	What foundational legal norm underpins the regulatory framework?
Administration	What organizational and human resource capacities exist to implement regulations?
Expertise	What technical and regulatory knowledge is included in the development and implementation of the framework?
Financing	What mechanisms for financing support the regulatory framework?
Participation	How are stakeholders engaged in the regulatory process?
Sponsor	What kind of leadership arrangements support the regulatory agenda?

In focused group sessions, participants interrogated the framework in more detail. They evaluated the tiers, proposed alternative formulations, added concrete country examples, and debated the trade-offs associated with different configurations. In doing so, they began to assemble shared illustrative cases that could, in future iterations, be developed to support users of the Compass. These exchanges generated cross-country learning, as participants compared how similar challenges had been addressed in Kenya, Rwanda and Uganda, often hinging on productive disagreement, as stakeholders used their national experiences to contest familiar narratives about what counted as feasible, legitimate or equitable regulatory design. Facilitators recorded suggested changes, which were then incorporated into a revised version of the framework. The updated DigiReg Compass was presented back to the full group for further discussion and validation (Table 4). The final Compass thus represents a co-created consensus-based tool that offers governments a structured yet flexible reference for regulatory design and provides a visible trace of the social learning that occurred during the workshop.

**Table 4:** DigiReg compass: Tiered institutional design features for digital health regulation

Feature	Tier 1	Tier 2	Tier 3
Norm	Guidelines and policies	Hybrid mix of policies/guidelines and selected statutory tools	Binding legislation/comprehensive regulations
Administration	Internal ministry units	Inter-ministerial coordination mechanisms	Independent or semi-autonomous regulatory agencies
Expertise	Knowledge integrated mainly through internal staff	Combination of internal staff and external consultants/advisors	Formal hybrid advisory bodies or standing expert committees
Financing	Reallocated existing funds	Donor or pooled/redistributed resources	Dedicated, recurrent budget lines for digital health regulation
Participation	Ad hoc consultations	Standing committees or periodic multi-stakeholder forums	Institutionalized councils with formal participatory mandates
Sponsorship	Support from technical officers/mid-level managers	Support from senior officials (for example, directors, permanent secretaries)	Cabinet-level or ministerial champions

### Data analysis

Our analysis of the qualitative material followed a reflexive thematic approach, structured around the research question “how can governments organize and use diverse forms of knowledge to make decisions about digital health under severe capacity constraints” and the six institutional design features underpinning the DigiReg Compass. We used the six institutional design features drawn from our draft evaluate framework as a primary organizing frame because our empirical focus was on how regulatory arrangements are configured and debated in practice. Through this process, we were attentive to how evidence travelled, how ideas were taken up or resisted and how interactional identities and practices may shape the understanding and appetite for co-creation. Instead of adding separate coding dimensions for knowledge mobilization and co-creation, we treated these bodies of work as cross-cutting lenses that guided how we interpreted patterns within and across the six institutional features.

Detailed notes from plenary and group discussions were reviewed to identify patterns in how participants interpreted documentary evidence, articulated problems and proposed regulatory options. We looked for instances of reflexivity, such as explicit acknowledgements of institutional limitations, negotiation of trade-offs such as choosing between statute and policy instruments, and for the ways in which participants situated proposed solutions within their national institutional and political contexts. We placed particular focus on learning moments, which are points at which participants revised prior assumptions, adopted or adapted concepts introduced by others or reformulated their positions considering comparative evidence.

We operationalized learning moments as instances where participants explicitly indicated a change in understanding, position or problem framing in response to evidence or others’ contributions. Empirically, these moments were often signalled by phrases such as “I had not seen it that way before,” “hearing this makes me think we should…” or “we used to assume X, but now I see we might need Y,” and were sometimes accompanied by laughter, pauses or gestures that we considered markers of reflection. For example, during a discussion on legal form, a senior official from one country remarked that comparing their guideline-based approach with Kenya’s Digital Health Act made them realize that Kenya had done a lot more work and had a certain level of political support that they struggled with internally and went on to express confidence in their current approach – a shift that we recognized as a significant reframing of their earlier position. In another breakout, a private-sector participant described how fragmented regulations hindered innovation because they did not know who to turn to for clarification or the channels through which to get that clarification on certain policies, which sometimes they were unsure whether they were compelled to follow or whether they were persuasive guidelines. This prompted another regulator to acknowledge that their own internal silos are part of the problem, and to revisit assumptions about where regulatory functions should sit.

These shifts in understanding, whether expressed as emerging consensus, clarified disagreement or recognition of contextual constraints, were treated as indicators of co-creation operating as a process of social learning and knowledge mobilization. In analysing the data, we therefore treated learning moments not only as shifts in stated positions but as instances where participants felt able to acknowledge gaps, revise prior assumptions or articulate discomfort within existing practices, often prompted by facilitation moves that invited self-assessment or explicitly validated uncertainty.

To strengthen interpretive robustness, we matched workshop data with the Phase 1 analysis. This allowed us to compare stakeholder interpretations with the formal policy record to identify where lived experience revealed gaps or tensions in existing frameworks and to see how participants drew on documentary evidence in arguing for particular design choices. The stepwise evolution of the DigiReg Compass provided an additional analytic lens. By tracking which features were retained, amended, or contested across rounds of revision, we could see which institutional design questions generated convergence, which remained unsettled and how the structure of the heuristic itself was collectively refined. Instead of treating the workshop as a static snapshot of stakeholder views, we read the material as a sequence of interactions in which understandings were tested, adjusted and sometimes abandoned. This enabled us to examine co-creation not only as a normative ideal but as an observable mechanism through which regulators and other actors organized, contested and generated knowledge for regulatory decision-making. To minimize researcher imposition, we prioritized participants’ own terms and framings, and summarized preliminary interpretations at the close of the dialogue to invite correction or clarification. This analytical strategy allowed us to capture both the substantive content of stakeholder deliberations and the dynamic process of collective reflection that underpins our conceptualization of co-creation as procedural innovation in digital health regulation.

## Results

### Plenary and focus group discussions

A central outcome of the co-creation workshops was the degree of reflexivity they fostered, particularly among policy-makers and regulators. Reflexivity here refers to the ability to critically interrogate one’s own assumptions, strategies and institutional practices. The workshops provided a rare opportunity for candid self-assessment, triggered both by cross-country comparisons and by constructive challenges from non-state actors. Regulators openly acknowledged gaps in their frameworks, such as limited adaptability, weak clarity, or inadequate enforcement, which often hindered innovation. For instance, one regulator, comparing their national approach with neighbours, remarked that seeing all three frameworks side by side forced them to admit that their framework is more aspirational than enforceable. These moments of recognition were frequently prompted by concrete examples from practice from private-sector and civil society participants, such as innovation consultants describing how overlapping approvals from three agencies delayed deployment of a basic telemedicine service.

The comparative element was especially catalytic. Kenyan regulators pointed to the enabling role of high-level political sponsorship in passing the Digital Health Act, prompting Ugandan participants to reflect on the absence of equivalent leadership in their context. Conversely, Uganda’s reliance on flexible guidelines without formal legislation resonated with Rwandan participants, who saw it as a feasible interim path. Such exchanges shifted the discussion from defending national approaches to collectively exploring alternative pathways and ways in which regulation is an epistemic project.

The workshops also served as a trust-building exercise. Initial defensiveness from regulators gave way to constructive engagement as they recognized shared goals with civil society and private innovators. Small-group discussions helped reframe challenges as shared responsibilities rather than zero-sum disputes. By the end of the process, dialogue was marked by collaborative language, culminating in joint achievements such as refining the DigiReg Compass. Participants increasingly used terms such as “we”, “our systems” and “shared responsibilities”, and began to co-author proposals on flip charts instead of defending country-specific positions. From a governance perspective, this reflects an important growth in institutional reflexivity, as participants left with both greater willingness and greater capacity to adapt regulatory approaches.

These dynamics were curated through facilitation strategies such as inviting regulators to speak about challenges, encouraging non-state actors to pose clarifying questions, periodically summarizing points of agreement and disagreement helped create a setting in which learning from each other was framed as co-creation. Participants explicitly linked their willingness to acknowledge institutional weaknesses to the perceived safety of the space, citing the closed-door format and the clear separation between the workshop and formal decision-making forums as enabling more honest dialogue than is usually possible in official consultations. The learning moments we observed depended not only on who was in the room but on design choices around invitations, staging and facilitation that signalled safety and legitimized humility and self-critique as part of regulatory practice.

Another key outcome was the explicit negotiation of trade-offs that typically remain implicit or decided behind closed doors. For example, one group mapped the advantages and risks of locating a digital health unit in a ministry versus an independent agency, productively interrogating and explicitly weighing political backing against agility and autonomy. Co-creation surfaced these tensions, allowed participants to weigh options openly and embedded them into the Compass as structured design choices. In sessions, facilitators asked stakeholders group to state what they would gain and lose under different arrangements, which made conflicting interests visible. A major debate concerned whether regulatory frameworks should be anchored in binding legislation or in softer instruments such as policies and guidelines. Kenya’s Digital Health Act illustrated the authority and public trust that legislation can generate, but also the political effort and rigidity it entails. Uganda’s guideline-based approach highlighted the speed and adaptability of softer instruments, but also the risks of fragmentation and weak compliance. Ugandan participants, for instance, described using guidelines to pilot some normative arrangements on interoperability with providers, with the explicit intention of later codifying successful elements in statute, while Kenyan participants reflected on how such sequencing might have eased the path to their Digital Health Act. Through dialogue, participants reframed the issue as a sequencing problem where flexible guidelines could serve as a stepping stone, with elements later codified into law once institutional readiness matured. This logic was incorporated into the Compass through a hybrid tier (Table 1).

A second trade-off lay between locating regulatory functions within ministries or creating new agencies. Internal units ensure alignment with broader health policy and are easier to establish but may lack focus and cross-sectoral authority. Independent agencies promise agility and dedicated expertise but require resources and risk siloization. Rwanda’s inter-ministerial model provided a middle path, pooling expertise across sectors. Consensus ultimately emphasized that mandate clarity and political backing mattered more than organizational form, a reflexive shift from structure to function. Moments of friction, such as disputes over whether to privilege legislation or guidelines, or over the appropriate location of regulatory authority, were analytically important because they made visible the underlying values and interests at stake and turned the workshop into a site of collective problem framing rather than simple consultation.

Financial sustainability was a shared concern. Existing health budgets provided pragmatic starting points, but all three countries acknowledged heavy donor reliance. Kenya’s experience underscored the risks of over-dependence, while discussions highlighted the value of iterative budgeting: starting with pilot funds, often donor-supported, then leveraging early results to justify incremental domestic investment. The Compass embeds this logic in its financing tiers by linking each stage to concrete sustainability actions.

Engagement was debated not in terms of “more is better”, but in terms of quality and institutionalization. While ad hoc consultations offered flexibility, they risked tokenism; permanent councils promised transparency and trust but required safeguards against politicization. Rwanda’s Health Data Governance Technical Working Group was cited as a workable middle ground. The Compass captures this by presenting participation as a continuum of institutionalization rather than a binary choice.

The workshops also underscored that effective regulatory design must be context sensitive. Kenya demonstrated how windows of opportunity, such as a new administration’s digital agenda, can accelerate reform. Rwanda highlighted the benefits of strong inter-ministerial coordination, while Uganda illustrated the value of incremental experimentation under resource constraints. By situating regulatory design within these national realities, participants challenged the universal applicability of “best practice” models and validated the need for adaptive, context-anchored strategies.

### The DigiReg Compass

The culmination of the workshop was the co-creation of the DigiReg Compass, a tiered heuristic designed to help governments organize and use knowledge about their regulatory systems when structuring or revising digital health institutional design (Table 4). Building on the initial evaluative framework, the Compass was iteratively revised through plenary debate and focus group input until participants agreed it was both descriptively accurate and practically useful. It organizes institutional design around six core features, each structured into three tiers. The tiers represent progressively greater formality and resource intensity, but they are not sequential stages. Rather, they map a spectrum of context-dependent choices and associated trade-offs, enabling policy-makers to reason systematically about options instead of searching for a single best practice. Each feature is anchored in a framing question (for example, “What kind of normative basis supports the framework?” or “What administrative structure implements the framework?”) and illustrated with concrete examples drawn from the three countries. In this way, the Compass captures not only formal arrangements but also the lived experience of how those arrangements function.

The Compass is designed as a decision-support heuristic rather than a blueprint. It went through three major iterations during the workshop. The initial version presented six features with binary choices; a second version, revised after the first day, introduced the tiered structure; and a third, final version renamed certain features for clarity and incorporated concrete country examples, which participants validated in the closing plenary.

Policy-makers begin with a diagnostic of their current context, reviewing existing legal frameworks, institutional arrangements, available expertise, financing capacity and political sponsorship. They then work through each feature, selecting the tier that best reflects both their baseline and their realistic direction of travel. For instance, a government relying on informal guidelines might opt for Norm, Tier 2 (hybrid), combining guidelines with targeted statutory provisions while deferring comprehensive legislation. Because tier choices must be made across all six features, the Compass guards against over-concentration on visible elements such as legislation while neglecting less obvious but equally critical dimensions such as financing or political backing. In their current implementation, these selections can be summarized in a table outlining strengths, risks and suggested next steps for each feature, effectively translating deliberation into a structured action agenda.

In the final plenary, participants validated the Compass as both a practical tool and a demonstration of co-creation in practice. Regulators noted that a similar heuristic could have provided structure and foresight in earlier policy processes; civil society participants emphasized its potential to make regulatory choices more transparent and to frame advocacy around feasible moves between tiers. Through discussion, participants refined elements of the framework, for example, renaming “Accountability” as “Norm” to clarify that the focus is on the type of instrument, and reiterated that tiers are context-sensitive options rather than rankings. The DigiReg Compass thus functions simultaneously as a product (a heuristic for regulatory design) and as a trace of the co-creation process itself, showing how structured, participatory dialogue can assemble fragmented perspectives into a shared decision-support tool that is immediately usable, adaptable and grounded in practitioner experience.

## Discussion

Our study set out to examine co-creation as a mode of organizing and mobilizing knowledge for digital health regulation in capacity-constrained settings. Our findings suggest that co-creation can function as a procedural innovation that structures how regulators and other actors assemble, interpret and re-use fragmented evidence, rather than simply as a participation exercise. Additionally, our findings suggest that the epistemic value of co-creation lies less in the production of agreement than in the structuring of contestation, as stakeholders are asked to interrogate evidence, name conflicts and live with residual disagreement while still moving towards workable regulatory choices. We locate these insights within debates on co-creation, collaborative innovation and decentred regulation, and draw out implications for governing digital health under constraint.

### Co-creation as knowledge mobilization and collaborative innovation

Much of the co-creation literature emphasizes multi-actor collaboration as a route to public-sector innovation but pays relatively less attention to *how* knowledge is organized and used within these processes [31]. Our findings contribute to filling that gap. The stakeholder dialogue was explicitly structured around a comparative documentary analysis and an evolving heuristic (the DigiReg Compass), which gave participants a shared reference point for making sense of dispersed legal texts, policy documents and experiential insights. Rather than treating co-creation as an open-ended conversation, the process made visible how different forms of knowledge were brought into play, tested and reconfigured.

The case resonates with accounts of collaborative innovation as a way of harnessing distributed innovation assets under conditions of complexity and resource constraint [27]. Regulators, clinicians, technologists and civil society actors did not simply express preferences but collectively interpreted comparative evidence and used it to reformulate questions about institutional design. The Compass, as a tiered set of options, crystallized this work into a knowledge translation device that participants could carry back into their own bureaucratic settings. While we cannot claim that this immediately transformed regulatory practice, it illustrates how co-creation can move beyond consultation to structure the circulation and uptake of knowledge in regulatory decision-making.

### Social learning and institutional reflexivity

The analysis also points to co-creation as a site of social learning and institutional reflexivity. Social learning theories stress that learning collective, emerging when diverse actors confront and negotiate differing problem framings [32]. The learning moments we noted, instances where participants explicitly revised their views considering others’ experiences or the documentary evidence, correspond to what double-loop learning theory describes as questioning underlying assumptions (Argyris and Schon [2]; Chatti et al. [6]). Such learning was visible both within and across countries – for example, participants from one jurisdiction reconsidering the importance of political sponsorship after hearing peers describe stalled initiatives without high-level backing, or participants revising assumptions about the inevitability of statutory law once trade-offs around flexibility and enforceability were surfaced. The stepwise co-creation of the DigiReg Compass made that learning durable by encoding it into tiered choices and framing questions. The Compass is thus a product of deliberation and a scaffold for ongoing institutional reflexivity, something closer to an experimentalist architecture for iterative adjustment than a fixed template [24].

### Decentred and networked regulation under constraint

Our findings also speak to debates on decentred regulation. Black [3] characterizes contemporary regulation as an activity dispersed across networks of public and private actors, with the state increasingly acting as a node or facilitator rather than a sole rule-maker. The co-creation process we observed fits this picture. Participants drew on the knowledge, agendas and capacities of non-state actors not only to generate ideas but also to gauge feasibility, foresee implementation barriers and identify potential allies. In a context of limited staff and technical resources, this form of networked engagement partly compensates for state capacity constraints by mobilizing what might be called network capacity: the expertise, legitimacy and practical support of actors beyond the core bureaucracy. At the same time, our data complicate more optimistic accounts of co-creation. While non-state participants influenced the structure and content of the Compass, government voices remained decisive when finalizing options and framing next steps. The process aligned with decentred regulation analytically but did not radically redistribute authority. This tension mirrors broader findings in the co-creation literature that governance traditions and power relations condition how far co-creation can move beyond advisory roles into genuine shared decision-making [30].

### Legitimacy, ownership and their limits

Participants described the Compass as “our tool” and reported that the process increased their sense of ownership over future regulatory trajectories. It is also important to note that some of the participants co-authored this article, signalling broad endorsement for the Compass. This echoes arguments that co-creation can enhance the democratic quality and perceived legitimacy of public solutions by involving those affected in cross-boundary collaboration [1]. In our case, relational gains, improved understanding between regulators and non-state actors, reduced defensiveness and explicit recognition of shared constraints were among the clearest outcomes.

However, we are cautious about overstating these effects. The workshops were time-limited and convened under favourable conditions. Sustaining trust and shared ownership requires repeated interaction over longer periods. Moreover, some voices were still under-represented, particularly end-users and those outside capital-city policy networks. Co-creation, as many have warned, can reproduce existing exclusions if participant selection and process design are not carefully managed [13]. Our findings support this caution, as the process broadened participation relative to closed-door drafting but did not fully resolve underlying representational imbalances.

Several limitations qualify our claims. First, the empirical base is narrow, based on a single multi-country workshop and associated content analysis. The sample of participants was purposive and skewed towards already engaged actors, and so perspectives from more marginalized groups in the health system, including patients and grassroots community organizations, are under-represented. Second, although we observed shifts in understanding and co-creation of the Compass, we did not follow subsequent regulatory processes. We therefore cannot assess how, or whether, the Compass was used in later decision-making, nor whether the observed learning translated into concrete institutional change. Third, the process was itself framed by the research team’s initial evaluative structure, which likely shaped the categories and trade-offs participants considered. This reflects a broader challenge in co-creation research of disentangling participants’ learning from the heuristics introduced by facilitators.

These limits point to a modest reading of our contribution. The study offers an existence proof that co-creation can be organized in ways that foreground knowledge mobilization and social learning in digital health regulation, and that such processes can generate usable heuristics such as the DigiReg Compass. It does not demonstrate superiority over alternative approaches, nor guarantee that co-creation will overcome entrenched power asymmetries or resource shortages. Future work could compare co-creation-based regulatory exercises with more conventional drafting processes, track the use (or non-use) of tools such as the Compass over time, and explore how different participant constellations, facilitation strategies and political contexts shape outcomes. An obvious next step is to curate a living repository of country examples linked to each tier of the Compass, to provide practitioners with concrete illustrations without prescribing a single model.

For practitioners, the key implication is pragmatic. Co-creation should be seen as a way to structure uncertainty and mobilize distributed knowledge under constraint, not as a cure-all. Used carefully, it can help regulators in LMIC settings move from fragmented, ad hoc engagement towards more systematic consideration of options and trade-offs. But its promise depends on attention to who is in the room, how evidence is presented and contested and whether there is institutional commitment to revisiting decisions as conditions change. Attending to contestation in this way helps move co-creation debates beyond celebratory accounts of harmony, and foregrounds the politics of whose knowledge is heard, which disagreements are legitimized and how unresolved conflicts are managed over time.

## Conclusions

The rapid expansion of digital health in LMICs has outpaced the development of coherent regulatory frameworks, leaving regulators to act under conditions of institutional fragmentation, resource scarcity and external pressure [21, 34]. Our study has examined one response to this challenge, which is co-creation as a structured mode of organizing and mobilizing knowledge for regulatory design. Rather than treating regulation as a one-off technocratic act, the co-creation process recast it as an ongoing, negotiated practice in which policy-makers, regulators, providers, technologists and civil society actors collectively interpret evidence, surface experiential insights and negotiate trade-offs [1].

Empirically, the workshops produced both a substantive artefact (the DigiReg Compass) and procedural effects. The Compass structures institutional design choices into tiered options across six features, providing policy-makers with a heuristic for reasoning systematically about what is feasible and desirable in their context, rather than reaching reflexively for imported best practice. Procedurally, the process generated relational benefits in the form of shared vocabulary, improved mutual understanding and a sense of joint ownership over future regulatory trajectories. In line with work on co-creation and collaborative innovation [22, 27], our findings suggest that such processes can partially compensate for capacity constraints by mobilizing networked expertise and legitimacy, and by making tacit assumptions and constraints discussable.

Theoretically, the study adds nuance to two lines of debate. First, it illustrates how co-creation can function as a modest form of experimentalist and decentred regulation, embedding opportunities for feedback and adjustment into the design phase and positioning policy-makers as orchestrators within wider knowledge networks rather than sole authors of rules [3, 24]. Second, it shows how social learning and reflexivity can be operationalized in practice, as learning moments were not simply changes in opinion but shifts in how participants framed problems and imagined institutional futures. The DigiReg Compass can thus be read as a device that stabilizes and carries forward collective learning, even if only within a relatively narrow circle of already-engaged actors.

However, our conclusions are necessarily modest. The empirical base is limited to one multi-country workshop. We do not track subsequent regulatory processes, and we cannot say whether the Compass was used or adapted over time. Power asymmetries persisted, with state actors ultimately decisive, and participation remained skewed towards institutional elites. Co-creation, as others have warned, can reproduce existing inequalities or degenerate into managed consultation if not accompanied by broader institutional changes and a commitment to reconvening, revisiting and revising [18]. So, co-creation should be seen less as a cure-all than as one pragmatic way to structure uncertainty and mobilize distributed knowledge under constraint.

For LMICs, the practical implication is cautious but concrete. Co-creation, anchored in comparative evidence and supported by simple heuristics such as the Compass, can help move from ad hoc, siloed engagement towards more explicit consideration of options, trade-offs and dependencies. Whether this potential is realized is as political as it is technical. Future research will need to follow regulatory trajectories over time, compare co-creation with more conventional rulemaking, and examine whose knowledge is systematically included or excluded. Nonetheless, our findings indicate that treating regulation as a collective learning project rather than a purely technical drafting exercise is both feasible and, in contexts of constraint, arguably necessary.

## Data Availability

All available data are contained in the manuscript.

## Declarations

### Reflexivity statement

This study is part of a Welcome Trust-funded multi-country study exploring various aspects of digital health regulation in sub-Saharan Africa. It aims to rethink digital health governance with critical stakeholders in the region. All the authors are from the African continent, except one person who is Afro-Caribbean, being the direct descendant of enslaved Africans from the Akan people of Ghana and the Yoruba of Nigeria. Four authors have prior experience conducting fieldwork in sub-Saharan Africa.

The authors include early-career and senior researchers and government officials affiliated with national governments and international institutions. Two authors are interdisciplinary scholars based in the Global North, and the remaining authors are health systems practitioners working at the intersection of digital health, regulation and governance in Kenya, Rwanda and Uganda. The sole woman author is the principle investigator (PI) of the project. We acknowledge that, while this may not be representative of gender inclusivity, it is representative of the current digital health ecosystem, which has a gender imbalance that privileges male leadership.

Our focus on co-creation reflects an intellectual and normative commitment to inclusive governance and co-authorship with the people who help create knowledge. Several of us have worked in projects supported by philanthropic and international agencies and are aware that these affiliations bring both opportunities and biases. We acknowledge the privileges conferred by our institutional affiliations and professional networks, which afford us visibility and influence in shaping discourses on digital health. Yet, we remain conscious of the asymmetries in power and voice between researchers, funders, governments and communities in LMICs. Engaging with stakeholders in Kenya, Rwanda and Uganda, we sought dialogue on iterative, context-sensitive processes leading to a new form of scholarship.

Finally, we see this paper as part of an ongoing learning process. Reflexivity, for us, means not only embedding equity and participation into research outputs but also questioning the processes, partnerships and institutional incentives that shape how knowledge is produced. We write with the aspiration of contributing to more adaptive, inclusive and accountable forms of governance while acknowledging our own roles within the systems we critique.
